# Strategic research agenda for biomedical imaging

**DOI:** 10.1186/s13244-019-0684-z

**Published:** 2019-01-28

**Authors:** The European Institute for Biomedical Imaging Research (EIBIR), Silvio Aime, Angel Alberich, Anja Almen, Owen Arthurs, Henryk Barthel, Olivier Clément, Michael Crean, Nandita de Souza, Florian Demuth, Marc Dewey, Vincent Dousset, Alejandro Frangi, Casper Garos, Xavier Golay, Peter Gordebeke, Matthias Günther, Horst Hahn, Monika Hierath, Christoph Hoeschen, Myriam Hunink, Hans-Ulrich Kauczor, Gabriel Krestin, Katharina Krischak, Georg Langs, Yan Liu, Luis Marti-Bonmati, Celso Matos, Ulrike Mayerhofer-Sebera, Jonathan McNulty, Kristoff Muylle, Michal Neeman, Wiro Niessen, Konstantin Nikolaou, Philippe Pereira, Anders Persson, Antonio Pifferi, Katrine Riklund, Andrea Rockall, Karen Rosendahl, Francesco Sardanelli, Steven Sourbron, Oliver Speck, Vincenzo Valentini, Pamela Zolda

**Affiliations:** The European Institute for Biomedical Imaging Research, Vienna, Austria

**Keywords:** Precision medicine, Preventive medicine, Radiology, Diagnostic imaging, Artificial intelligence

## Abstract

This Strategic Research Agenda identifies current challenges and needs in healthcare, illustrates how biomedical imaging and derived data can help to address these, and aims to stimulate dedicated research funding efforts.

Medicine is currently moving towards a more tailored, patient-centric approach by providing personalised solutions for the individual patient. Innovation in biomedical imaging plays a key role in this process as it addresses the current needs for individualised prevention, treatment, therapy response monitoring, and image-guided surgery.

The use of non-invasive biomarkers facilitates better therapy prediction and monitoring, leading to improved patient outcomes. Innovative diagnostic imaging technologies provide information about disease characteristics which, coupled with biological, genetic and -omics data, will contribute to an individualised diagnosis and therapy approach.

In the emerging field of theranostics, imaging tools together with therapeutic agents enable the selection of best treatments and allow tailored therapeutic interventions.

For prenatal monitoring, the use of innovative imaging technologies can ensure an early detection of malfunctions or disease.

The application of biomedical imaging for diagnosis and management of lifestyle-induced diseases will help to avoid disease development through lifestyle changes.

Artificial intelligence and machine learning in imaging will facilitate the improvement of image interpretation and lead to better disease prediction and therapy planning.

As biomedical imaging technologies and analysis of existing imaging data provide solutions to current challenges and needs in healthcare, appropriate funding for dedicated research is needed to implement the innovative approaches for the wellbeing of citizens and patients.

## Key points

- Innovative biomedical imaging plays a crucial role in personalised medicine, disease prevention and therapy monitoring
- Innovative biomedical imaging technologies and analysis of existing imaging data provide solutions to current challenges and needs in healthcare
- Appropriate funding for biomedical imaging research is necessary to implement innovative approaches addressing citizens’ and patients’ needs

## Introduction

Medicine and healthcare in general are experiencing a major change, moving from reactive to proactive approaches by providing predictive, preventive and personalised medical solutions for the individual patient. Particularly, the concept of personalised medicine is promoted by the European Commission as it addresses the challenges of ineffective treatment and rising healthcare costs with patient-centred prevention and treatment plans. Personalised and precision medicine has a huge growth potential, which provides Europe’s healthcare industry with the opportunity to further expand its leading position, contributing to economic growth and job creation.

Furthermore, Europe needs research, innovation and applications for the benefit of all. In this context, areas of research have to be prioritised and research programmes should focus on high-impact outcomes [1]. In its recently launched work programmes [2], the European Commission has identified a number of healthcare challenges, e.g. the rising costs of health and care or the influence of external environmental factors including climate change on health, that need to be addressed by Europe’s research community to develop sustainable solutions that will help to overcome those challenges, improve the treatment of patients and lead to better health and wellbeing for European citizens. The development and adoption of good-quality and safe e-services can ensure that the health systems of Member States become more efficient and sustainable [3].

However, as stated in the European Council’s conclusions on Health in the Digital Society [4], new opportunities are arising from big data and improved data analytics capabilities, yet barriers to scaling up the potential in digital health and connected care still need to be overcome. These include the dominance of data silos, lack of interoperability and of common standards for measuring clinical and patient reported outcomes, and limited access and use of large databases for research and innovation purposes. Moreover, a lack of funding and financial incentives exists, which together with market fragmentation in the EU and across the spectrum of services further impedes innovation. Progress in implementing data-driven digital solutions in healthcare requires rigorous validation and testing of new solutions’ clinical effectiveness.

It is the aim of this Strategic Research Agenda for Biomedical Imaging to:

- Identify the current challenges and needs in medicine and healthcare
- Illustrate how biomedical imaging can help address these challenges
- Stimulate dedicated research funding efforts

Innovative imaging technologies are now allowing researchers to visualise, characterise and measure biological and molecular phenomena with a hitherto unattainable precision. For the first time in history, hallmark processes of human disease, such as tumourigenesis or molecular deposits, can be visualised in real time. Imaging technologies and computerised radiomics allow breakthrough discoveries and their application in healthcare and are thus the central tool driving fundamental research in biomedicine. Technological innovation in biomedical imaging has been growing in the last decades and significant advances in the research fields of photonics, physics, chemistry and computing are often quickly translated into instrumentation or procedures for biomedical imaging. This may accelerate medical advances, but also calls for a need to adapt existing and to implement new methods and care pathways for better patient outcomes.

Biomedical imaging is a key component in personalised medicine [5]:

- Personalised prevention will rely on non-invasive or minimally invasive image-based screening programmes [5]
- Structural, functional, physiological and molecular imaging biomarkers affect decisions on the type and intensity of treatment [5]
- Treatment response assessment with imaging biomarkers will improve personalised and targeted treatment [5]
- Imaging-supported non- or minimally invasive intervention integrates precision diagnosis and personalised treatment [5] and will contribute to value-based healthcare [6]

Moreover, biomedical imaging is a forerunner in the digital transformation of healthcare and a leading discipline in the adoption of new innovative solutions like deep learning and artificial intelligence. Digital health is essential for ensuring that breakthroughs or innovations in clinical research are translated into practice cost-effectively.

The European Institute for Biomedical Imaging Research (EIBIR) is a non-profit organisation with a network of more than 80 leading European research institutions and 11 European scientific societies related to biomedical imaging as shareholders. EIBIR is committed to coordinate and support the development of biomedical imaging technologies and the dissemination of knowledge with the ultimate goal of improving the diagnosis, treatment and prevention of disease. It supports research networking activities and plays a key role in spreading good practice and promoting common initiatives and interoperability in the field of biomedical imaging research (www.eibir.org).

EIBIR, together with its members and shareholder organisations, has identified five main challenges which biomedical imaging can help overcome:

- **Challenge 1**: Meeting the healthcare demands of Europe’s population through personalised disease prevention and therapy monitoring facilitated by medical imaging
- **Challenge 2**: Developing new disease-specific, targeted and image-guided therapies
- **Challenge 3**: Contributing to a healthy start by delivering early and improved information on foetal health and prenatal growth, and preventing the development of anomalies using advanced imaging technologies
- **Challenge 4**: Providing accurate assessments of the impact of lifestyle and environmental factors on health supported by medical imaging
- **Challenge 5**: Making Europe the world leader in machine-learning and artificial intelligence in medical imaging by exploiting existing data and expertise to implement digital solutions after rigorous clinical validation.

## Challenge 1: Meeting the healthcare demands of Europe’s population through personalised disease prevention and therapy monitoring facilitated by medical imaging

### Precision medicine has emerged as a novel healthcare paradigm during the past decade

The approach for risk prediction and disease prevention taking into account individual gene variability as well as environmental and lifestyle factors involves the integration of information from multiple sources to achieve patient population stratification and provide more specific diagnoses, focused treatment and better response assessment. Deep phenotyping in combination with genetic, biochemical and physiologic biomarkers allows prediction and early assessment of disease. The emerging field of radiomics links genotypic information to phenotypic disease manifestations using imaging. Radiomics can greatly contribute to patient-tailored prevention and care.

Special attention should be devoted to the development of safe and more specific contrast agents, and the optimisation of acquisition procedures to the characteristics of the treated patient/pathology enabling precision radiology.

Clinically oriented research on quality and safety of medical imaging is an essential aspect when advancing precision radiology. In 2017, five EIBIR shareholder organisations established the European Alliance for Medical Radiation Protection Research (EURAMED; www.euramed.eu) and published a strategic research agenda for radiation protection in medicine [7], highlighting the research needs in this field and encouraging sustainable European efforts.

Imaging biomarkers are objectively measurable indicators of biological processes and can be used for the prediction of patient outcome (regardless of therapy) and response to specific therapies, and for response assessment (monitoring) [8]. The use of imaging biomarkers is non-invasive—a clear advantage over invasive biopsies [8]. Another advantage is the assessment and quantification of cellular targets for the entire disease burden, avoiding sampling errors that can occur with heterogeneous expressions, as well as the potential for serial studies of the in vivo effects of a drug or action on the target [8]. In addition, imaging biomarkers can unravel specific localised biological pathways and enable therapeutic targeting of such specific biomolecular mechanisms [8].

Joint efforts between research institutions and technology vendors should lead to the implementation of standards in image acquisition, production, archiving and distribution, and ethical use of sensitive data. Such approaches are a prerequisite for sharing large imaging data sets across Europe and for the discovery, adoption and wider use of predictive, prognostic and diagnostic imaging biomarkers. The European Imaging Biomarker Alliance (EIBALL) is a joint initiative operating in conjunction with EIBIR and in collaboration with the Quantitative Imaging Biomarkers Alliance (QIBA) and the European Organisation for Research and Treatment of Cancer (EORTC) for the development and validation of new imaging biomarkers.

#### Examples addressing Challenge 1

##### Example 1: BRCA1/2 carriers, enhanced imaging-based monitoring for breast and ovarian cancer (conventional mammography vs. contrast-enhanced MRI)

**Fig. 1:**
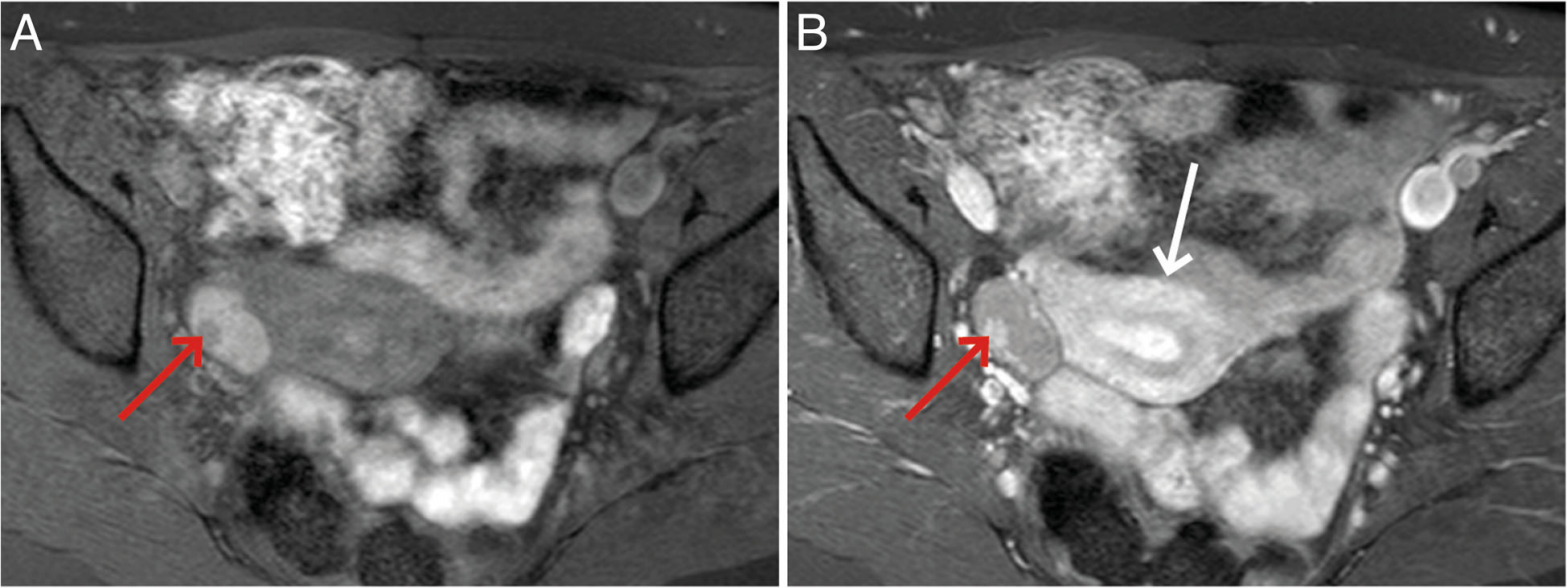
MRI scan of early-stage 1A high-grade serous ovarian cancer. The BRCA1-positive patient was identified through ultrasound screening, confirmed by MRI. The right ovary contains as cyst with an enhancing nodule (red arrows) demonstrated on the T1 fat-saturated image before (**a**) and after (**b**) contrast administration. The nodule enhances brightly, similar to the adjacent uterine myometrium (white arrow). Detection of ovarian cancer at this early stage is associated with a significantly improved 5-year survival rate compared with later stages of disease (courtesy: A. Rockall)

Persons carrying mutations in the BRCA1/2 gene are more likely to develop breast cancer (men and women), ovarian cancer in women and prostate cancer in men. Genetic screening of high-risk families allows the possibility of early detection of disease or indeed prevention. There is now compelling evidence that MRI is the most appropriate screening test for breast cancer in BRCA1/2 carriers. However, screening programmes are predominantly based on mammography. Future screening programmes are needed to fully evaluate the position of MRI in screening women in high-risk categories. In ovarian cancer (Fig. 1), but also in other cancers such as prostate cancer, biomarkers of early disease in BRCA carriers, such as circulating DNA, are highly likely to form the basis of future screening in BRCA carriers in conjunction with imaging tests, including ultrasound (US) and MRI. Imaging has been highly successful in determining the likelihood of malignancy in ovarian and prostate masses. Research into translating this to screening practice is of utmost importance, particularly in relation to early detection of disease and treatment stratification by combining circulating and imaging biomarkers.

##### Example 2: Development of arterial spin labelling as a brain imaging biomarker

**Fig. 2:**
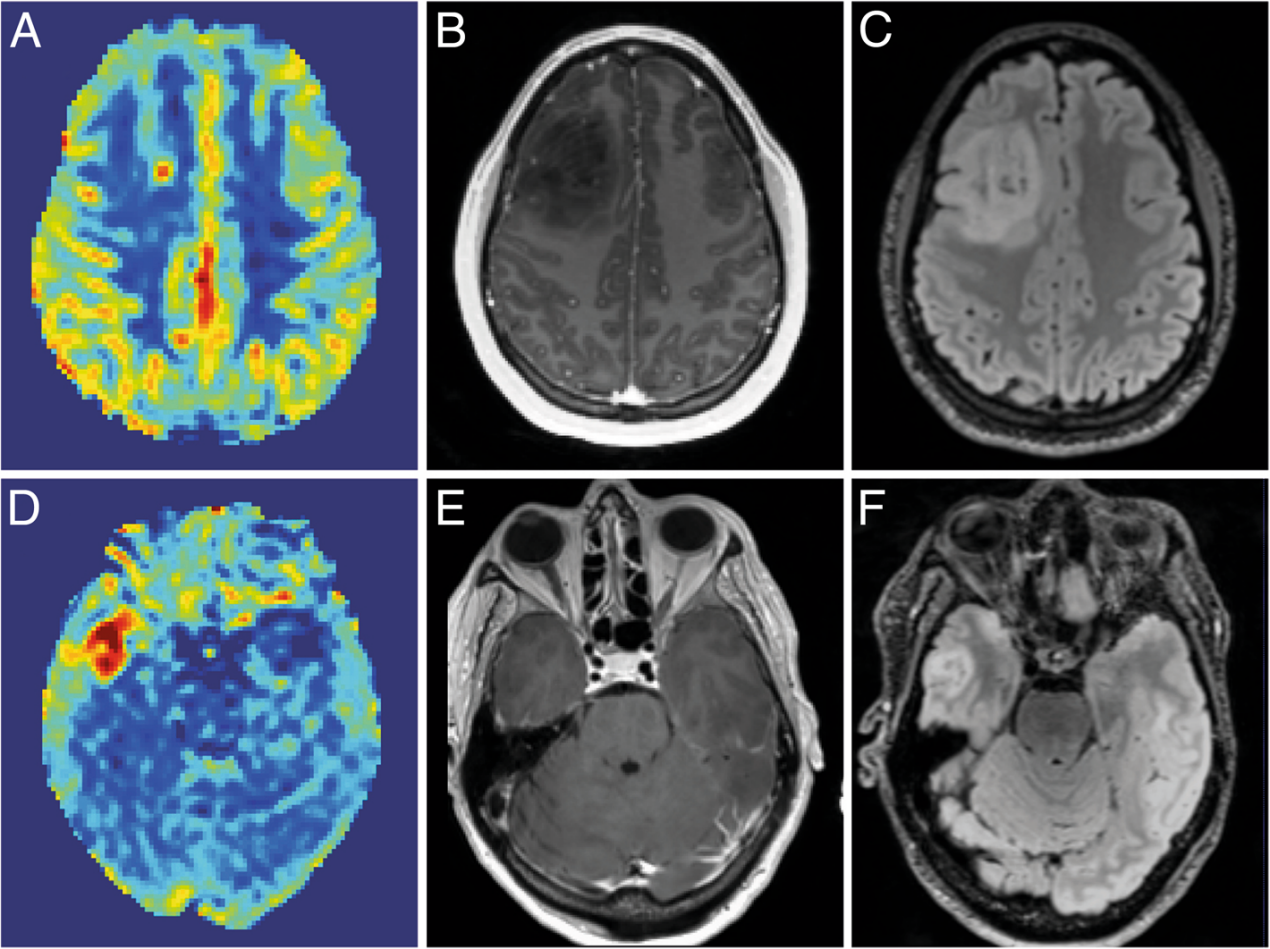
**a**–**c** Example of a 24-year-old female patient, with oligodendroglioma (grade II) in the left frontal lobe. **a** Cerebral blood flow, imaged by ASL. Red indicates high blood flow. **b** T1-Gd and **c** FLAIR. **d**–**f** Example of a 63-year-old female patient with GBM (grade IV) in the right temporal lobe. **d** Cerebral blood flow, imaged by ASL. Red indicates high blood flow. **e** T1 post Gd. **f** FLAIR. A clear difference in tumour blood flow can be seen between both patients (courtesy: A. Alsaedi, S. Bisdas)

Arterial spin labelling (ASL) to measure perfusion has been at the forefront of physiological imaging without the use of contrast agents for more than three decades now. In spite of its obvious advantages in numerous diseases, such as brain tumours (Fig. 2), dementia or stroke, ASL has remained underused. In particular, it has been shown [9] that relative tumour blood flow values can be used as an independent biomarker of glioma grading, while the same biomarker can accurately distinguish pseudo-progression from true progression, thereby providing a very important tool for therapy monitoring [9]. In 2014, a landmark paper established a roadmap for all manufacturers and users of this technology. Based on this, an on-going effort is taking place to establish a jointly developed EIBALL-QIBA profile detailing guidelines on how to use ASL in clinical practice. As part of this initiative, a commercial ASL calibration phantom was recently launched, together with an online calibration tool. For ASL to finally become accepted, it will be critical for new studies to show the repeatability and reproducibility of the assessment of perfusion over time and its increased usefulness in many clinical neurological affections.

## Challenge 2: Developing new disease-specific, targeted and image-guided therapies

### Biomedical imaging is an indispensable tool in personalised medicine providing information on localisation, extent, homogeneity and aggressiveness of disease

Diagnostic imaging procedures are increasingly used to support individualised and targeted treatment and represent a mainstay of the progress of personalised medicine [10]. This may include:

- The use of an imaging technology to monitor a therapy [10]
- The administration of an imaging agent acting as a drug surrogate to predict the biodistribution of the drug [10]
- The co-administration of both a drug and an imaging agent to assess in real time the delivery/release of the therapeutic agent at the pathological site [10]
- The use of an imaging agent to guide surgeons or interventional radiologists in a (minimally) invasive procedure [10].

Recent advances in molecular biology proved that drug response is often a result of different genetic alterations, but can also arise from micro-environmental and microbiome exposure. Altered environmental and genetic factors can facilitate cancer progression and may also influence drug effectiveness, if gene mutations are involved in drug metabolism. A better understanding of the disease biology as well as molecular changes and altered signalling pathways will enable the identification of patients who may benefit from such treatments. It will catalyse the development of targeted therapies which counter the influence of specific molecular drivers contributing to the development and spread of disease, providing the foundation of precision medicine.

Theranostics is a novel emerging field of treatment in which diagnostic imaging tools coupled with therapeutic agents allow precise targeting of a disease at a molecular level. Theranostics enables tailored interventions, further personalising healthcare practices to individual patients for whom a standard therapy is not suitable. However, more work is needed to identify novel theranostic combinations. New diagnostic tracers can be tailored to the specific needs of stratified patient groups or to rare diseases for which other therapeutic approaches are not yet available.

For most currently available radionuclide therapies, a fixed amount of radioactivity is administered, regardless of patient weight or body surface area. Although these empirical dosing methods are well-established, safe and effective, the theranostic approach offers the possibility for an upfront evaluation of the biodistribution, quantification and calculation of the absorbed dose to the target volumes and critical organs. The development of a personalised dosimetry-based approach might further improve the outcome and cost benefit of radionuclide therapies. Although dosimetry is essential for the development of radiopharmaceuticals, its potential and clinical use to tailor the administered amount of radioactivity for each individual patient still needs to be established and evaluated by randomised controlled trials comparing dosimetry-based versus fixed-activity approaches.

#### Examples addressing Challenge 2

##### Example 3: Theranostics for treatment of metastatic pancreatic cancer

**Fig. 3:**
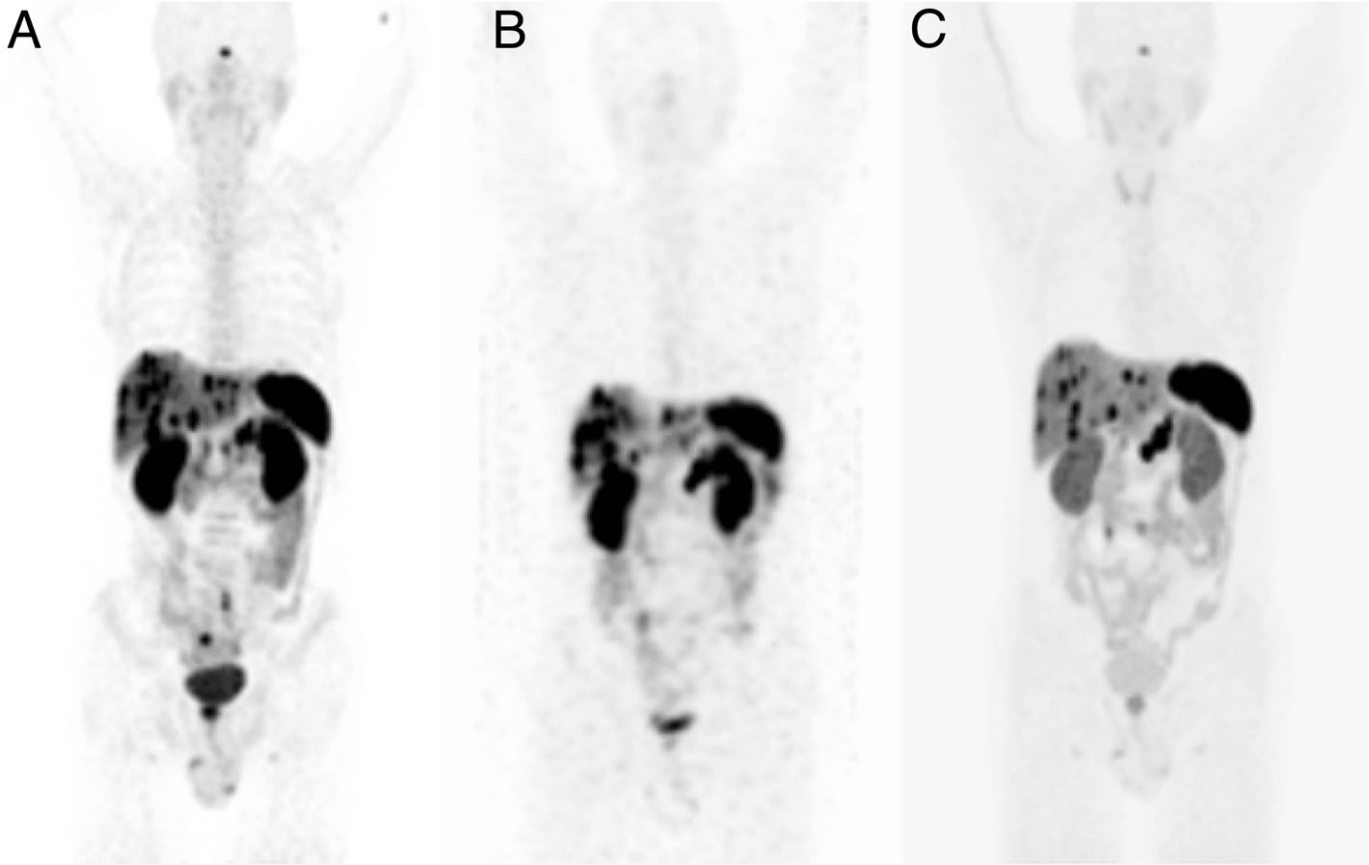
Theranostics in a patient with a metastatic pancreatic neuroendocrine tumour. **a** ^68^Ga-Dotatate-PET scan for staging. **b** SPECT evaluation after treatment. **c** End-of-treatment ^68^Ga-Dotatate-PET scan (courtesy of J. Kunikowska, Warsaw)

Figure 3 shows an example of theranostics in a patient with a metastatic pancreatic neuroendocrine tumour. In the first panel, staging with ^68^Ga-Dotatate-PET shows a high expression of somatostatin receptors in the pancreatic tumour, locoregional lymph nodes and liver metastases. Panel b shows evaluation with SPECT after treatment with ^177^Lu-Dotatate, confirming adequate targeting of the tumour sites. The end-of-treatment ^68^Ga-Dotatate-PET scan (panel c) shows a partial remission, particularly of the metastases in the liver. A recent phase III study shows that treatment with ^177^Lu-Dotatate results in a significantly higher response rate and longer progression-free survival than high-dose octreotide among patients with advanced midgut neuroendocrine tumours. A first interim analysis shows preliminary evidence of an overall survival benefit as well [11]. Similar theranostic approaches, such as ^68^Ga-/^177^Lu-PSMA in prostate cancer, are currently under evaluation in clinical trials with promising preliminary results.

##### Example 4: Cell recruitment to detect and target cancer for therapy

**Fig. 4:**
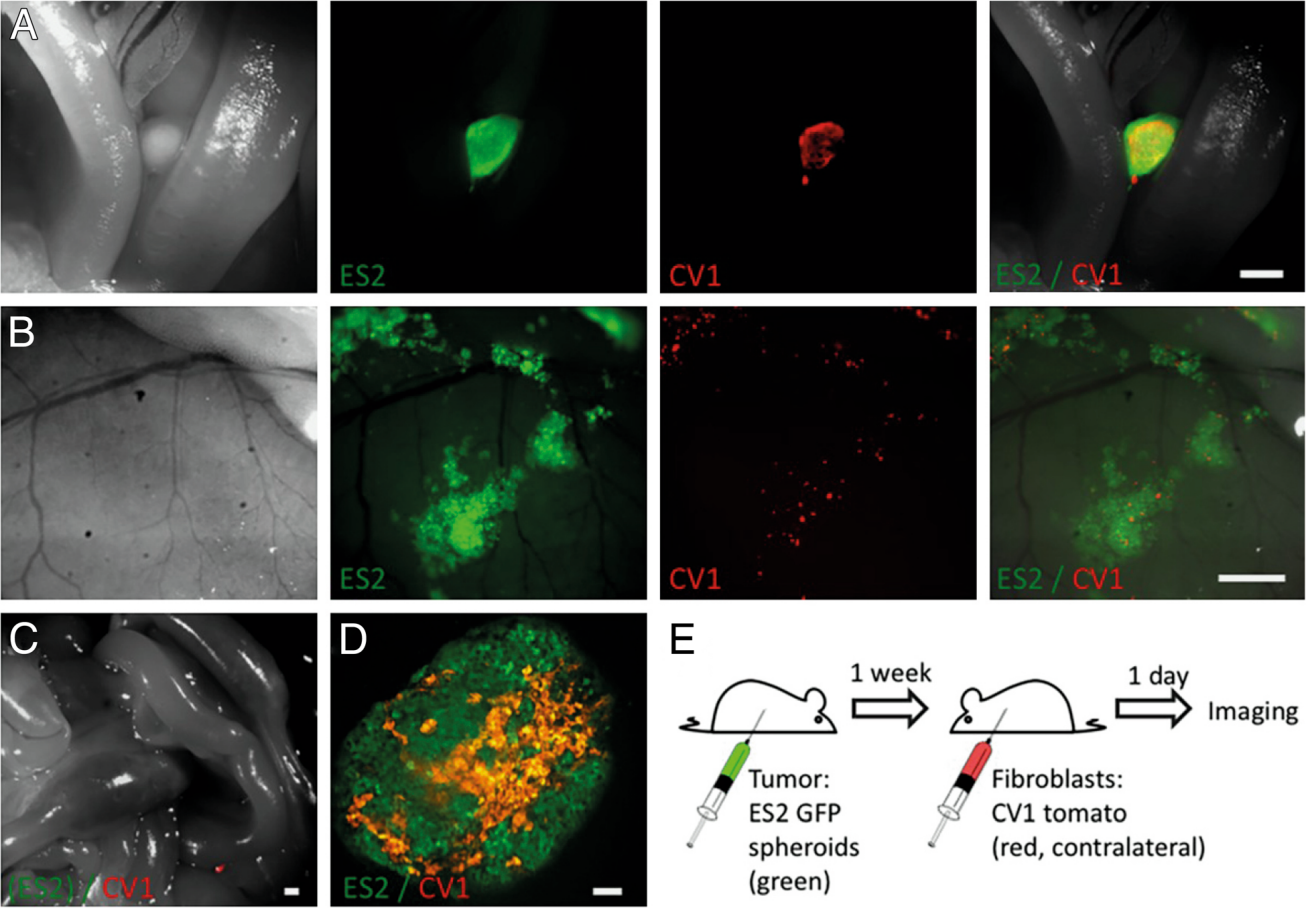
Fluorescence imaging of a mouse showing fibroblast (red) migration from injection site to location of tumour cells (green). (**a**) Fibroblast recruitment to tumour injection site next to intensine. (**b**) Fibroblast recruitment to tumour patches attached to the peritoneal wall. (**c**) Fibroblast scattered in the abdomen of a control mouse without tumour injection. (**d**) High resolution image of fibroblasts recruited to a tumour. (**e**) experimental design (green) [12]

Recent studies have shown that it is possible to use fibroblast recruitment as a tool for detection of cancer and targeted therapy [12]. Cells like fibroblasts labelled with a fluorescent reporter can be used as beacons for guidance of biopsy or surgery, as well as for therapy (Fig. 4). This figure illustrates study results that show fibroblasts migrate towards cancer cells. Additionally, a therapeutic effect could be seen in this study. Delivery of the labelled cells stopped the production of ascitic fluid in the abdomen in an orthotropic model of ovarian cancer metastasis and prolonged survival in mice.

## Challenge 3: Contributing to a healthy start by delivering improved information on foetal health and prenatal growth and preventing development of anomalies using advanced imaging technologies

### Biomedical imaging plays an important role in assessing foetal and prenatal health

The placenta is one of the most important organs influencing not just the health of a woman and her foetus during pregnancy, but also the lifelong health of both. When it malfunctions, serious problems such as gestational diabetes, preterm labour and stillbirth can occur. Placental dysfunction may also lead to health problems later in life for both mother and child. The early diagnosis of foetal malformations and growth anomalies has the potential to improve foetal prognosis by granting a treatment plan and by making specialist units and treatments from birth accessible. The diagnostic accuracy of ultrasound (US), the currently most-used imaging modality, is however limited.

Novel imaging technologies should be used in clinical settings. The use of advanced US tools (e.g. elastography), optoacoustic imaging and quantitative MRI can be essential for the assessment of placenta function, transport of nutrients, oxygen, and therapeutics across the placenta while also detecting aberrations in foetal development from implantation to birth. Studies should focus on maternal and foetal risk models including genetic, infectious and environmental influences for disease prevention.

Enhanced prenatal monitoring has the potential for early detection and intervention to reduce maternal and foetal morbidity and mortality. Furthermore, among the diseases and anomalies encountered in high-risk patients during pregnancy, e.g. preeclampsia, intrauterine growth restriction, multi-foetal pregnancies, increased maternal age and infection (e.g. ZIKA virus) can be diagnosed and monitored by imaging. In general it can be expected that further advances in imaging hold a striking promise for the study of the placenta and the foetus and emerging technologies can be translated to other organs and open new research avenues for the benefit of human health.

#### Examples addressing Challenge 3

##### Example 5: Foetal and perinatal imaging (US and MRI)

**Fig. 5:**
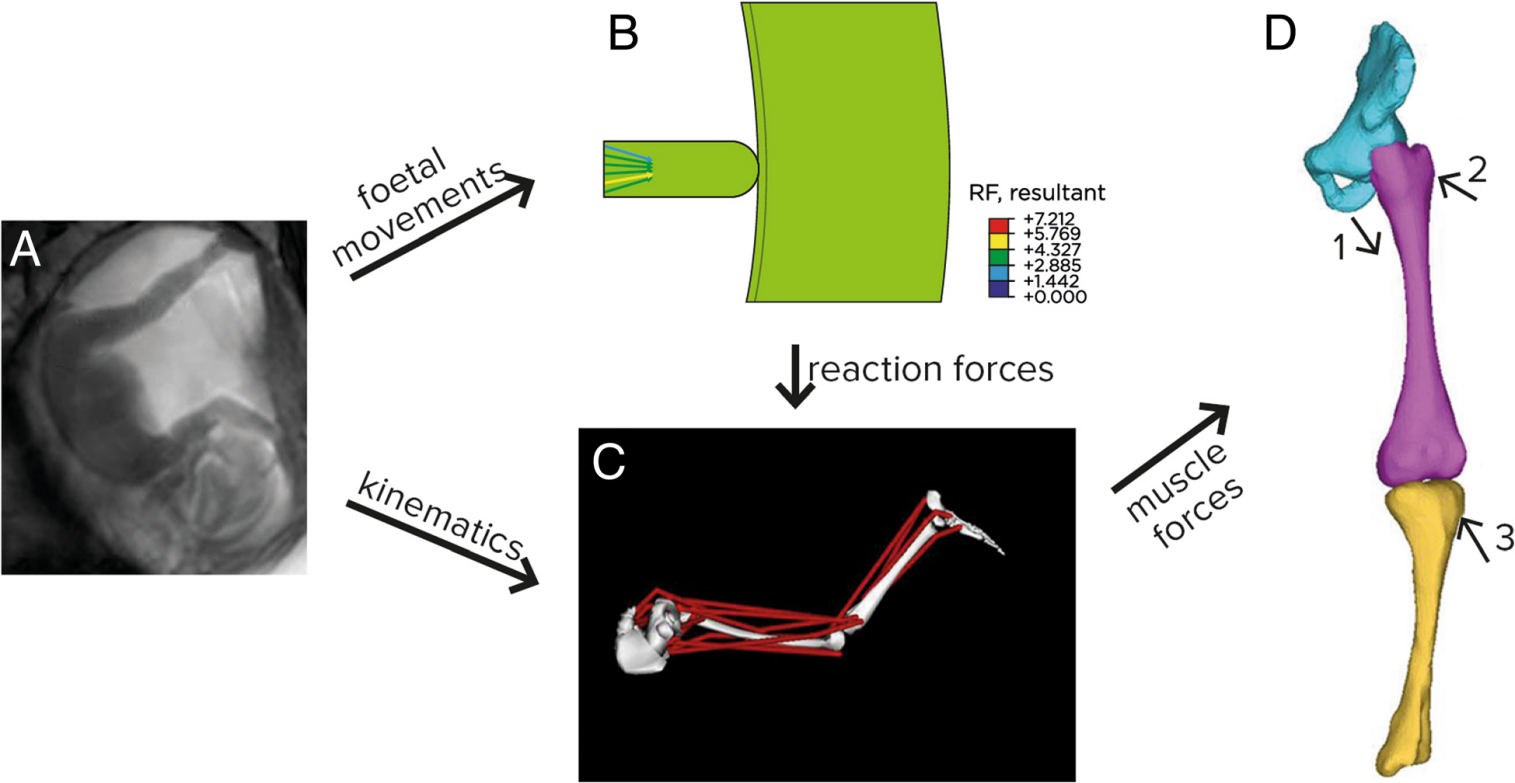
Computational pipeline for developing stress modelling of foetal movements. Foetal joint movements are tracked in utero (**a**), with finite element modelling of reaction forces (**b**) combined with musculoskeletal modelling to predict muscle forces (**c**) which are then applied to finite element models of foetal geometries (**d**). Adapted from [21]

Imaging techniques for prenatal imaging should be developed to identify in utero foetal growth disturbance, including foetal growth restriction and movement disorders. Normal foetal movement in the womb is critical to normal musculoskeletal development, and novel imaging strategies can play a critical part in diagnosis and monitoring of these diseases. For example, a spectrum of techniques including US elastography, micro CT, quantitative CT and MRI can be used to develop models of normal foetal movement (Fig. 5), against which diseases such as developmental hip dysplasia and foetal akinesia can be assessed. Early diagnosis and recognition will help reduce the huge societal and financial impact these diseases have on chronic musculoskeletal conditions in childhood, in combination with neonatal screening programmes for, e.g. hip dysplasia. The understanding and modelling of relationships between concurrent developments such as movement, skeletal system and the brain are key to the exploitation of perinatal imaging.

##### Example 6: BOLD imaging of the placenta

**Fig. 6:**
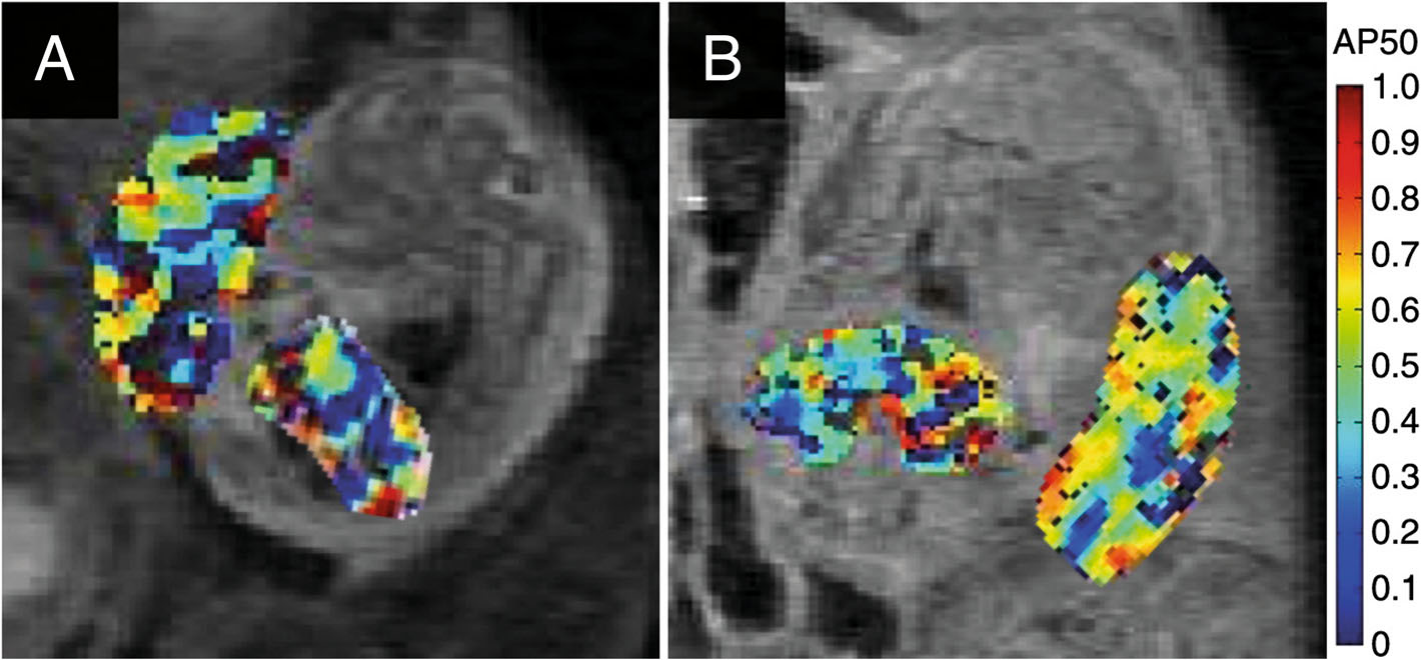
BOLD imaging analysis of pregnant mice. Representative spatial distribution maps of the oxygen-haemoglobin dissociation inside the placenta and foetal liver on days 14.5 (**a**) and 17.5 (**b**) show distribution and variability [22]

While structural imaging of the foetus and the placenta are technically established, though not widely available, the assessment of placental function during pregnancy is currently a field of research. The gas and nutrient exchange between the mother’s and the foetus’ blood circulation via the placenta is essential for the development of the foetus. Early delivery is recommended for placental dysfunction but has to be weighed against the risks of preterm birth. Oxygen exchange is a most relevant function of the placenta and, due to the high blood volume methods adopted from functional brain imaging with MRI, has been proposed to evaluate placental function (Fig. 6). During an oxygen challenge, the change in blood oxygen level-dependent (BOLD) signal is measured. In particular, the time constant of the related oxygen change on the foetal blood circuit side of the placenta has been shown in early studies to be related to birth weight or foetal brain and liver volumes. Biomarkers derived from US measurements of the placenta and foetus have not correlated similarly well to postnatal pathologic assessment of the placenta as BOLD MRI variations. The quantitative determination of placental oxygenation remains a challenge since the BOLD signal depends on many parameters that are difficult to assess non-invasively.

## Challenge 4: Providing accurate assessment of lifestyle and environmental impact factors on health supported by medical imaging

### The impact of lifestyle and environmental factors can be assessed by medical imaging

According to the WHO [13], 60% of the factors contributing to individual health and quality of life are correlated with lifestyle. Millions of people follow an unhealthy lifestyle and encounter illness, disability and even death because of it. Problems such as metabolic diseases, joint and skeletal pathologies, cardiovascular diseases, hypertension and violence can be caused by an unhealthy lifestyle. The relationship between lifestyle and health should be considered as established. Also the interaction between human health and the environment has been extensively studied and environmental risks have been proven to significantly impact human health, either directly by exposing people to harmful agents, or indirectly by disrupting life-sustaining ecosystems. Although the exact contribution of environmental factors to the development of disease and death cannot be precisely determined, the WHO has estimated that 13 million deaths annually are attributable to preventable environmental causes [14].

The timely use of diagnostic imaging tools as a preventive measure, followed by lifestyle changes, can prevent disease development [15]. Due to the increasing prevalence of obesity and its metabolic manifestations, it is important to obtain patient-specific quantitative body composition knowledge to predict and prevent disease [16]. The role of metabolically active tissues such as brown adipose tissue needs to be studied with new imaging methods to measure both mass and metabolic activity, enabling accurate assessments of energy turnover under normal and stimulated conditions. The identification of management strategies in cardiovascular diseases (CVDs) is of utmost importance, as the WHO projected that in 10 years from now more than 23 million people will die annually due to CVDs.

The use of biomedical imaging is essential due to its strategic advantages in diagnostic and therapeutic decision-making and as it delivers inputs for different stages of disease management, including prediction, screening, early diagnosis, staging, prognosis and follow-up. Deep phenotyping using imaging in combination with information acquired from wearable sensors has the significant potential to monitor subjects and patients during daily activities, providing information on external factors influencing biological processes at different levels.

#### Examples addressing Challenge 4

##### Example 7: Body fat imaging with quantification

**Fig. 7:**
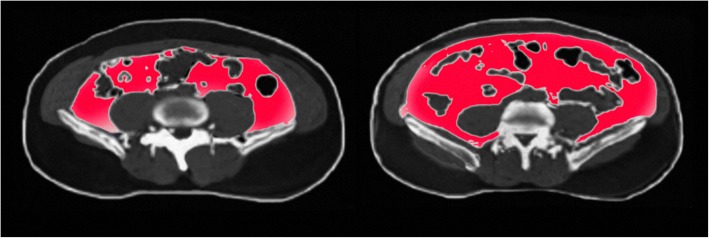
Men matched for the same BMI and total body fat: Differing ‘adiposity phenotypes’ regarding visceral obesity and subcutaneous obesity and with different risk profiles (courtesy: A. Persson)

The increase in obesity and metabolic-related diseases is one the challenges for global healthcare systems. Fat accumulation in organs and muscles is a strong biomarker of diabetes, the metabolic syndrome and obesity. A comprehensive understanding of the volume, distribution and quality of fat and muscles in the human body is important for diagnosis and treatment of illness. There is a need to develop automated segmentation and quantification of muscle and fat tissue volumes in the body (Fig. 7) which can be addressed by MRI. This technique has the potential to foster links between endocrine and cancer MRI disciplines and to develop biomarkers of morbidity in cancer survivors and links between disease entities such as body fat, diabetes, cancer and sarcopenia. In addition, the amount of subcutaneous and visceral adipose tissue in the abdomen plays an essential role in the determination of health risks.

##### Example 8: MR phenotyping in diabetes and cardiovascular diseases

**Fig. 8:**
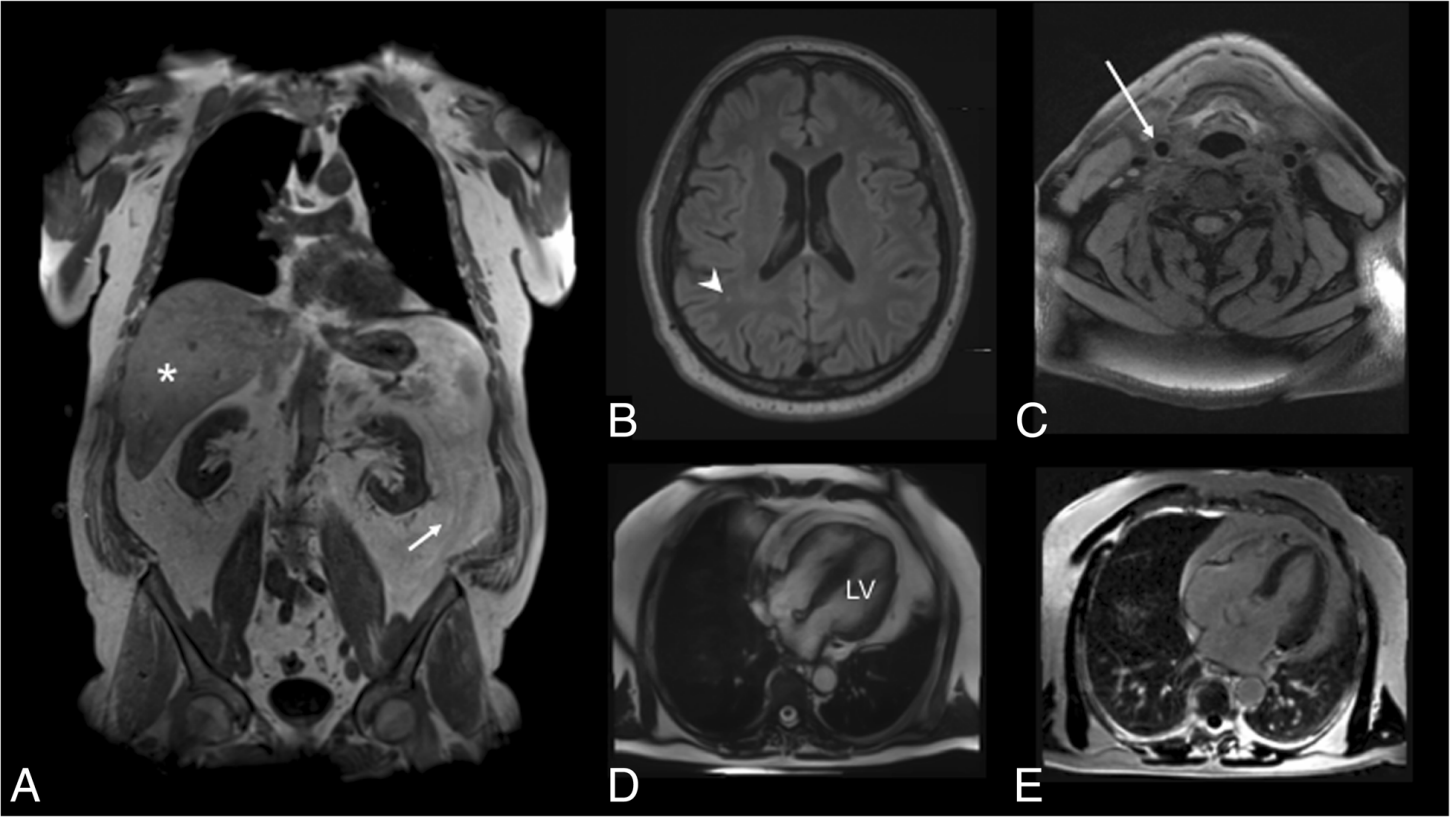
Imaging findings in a 61-year-old male indicating extensive subclinical disease burden. **a** Two-point DIXON T1-weighted sequence for the assessment of visceral adipose tissue volume from the femoral head to the cardiac apex (arrow) indicating high levels of fat as well as hepatic proton density fat fraction (asterisk, measured on multi-echo VIBE T1-weighted sequences). **b** Fluid-attenuated inversion recovery sequences demonstrating mild white matter lesions (arrowhead). **c** Atherosclerotic carotid plaque was determined on black-blood T1-weighted fat-suppressed sequences in the common carotid artery (arrow), the carotid bulb and the proximal internal carotid artery. **d** Cine-SSFP sequences were evaluated for the calculation of volume and mass left ventricle (LV). **e** late gadolinium enhancement was detected on fast-low-single-shot inversion recovery sequence four-chamber view [23]

Imaging plays a highly promising role in individualised risk assessment, particularly regarding population-relevant diseases such as diabetes mellitus and hypertension. In subjects with established diabetes or hypertension, there is strong evidence that the level of detectable subclinical disease burden, such as an altered myocardial perfusion and/or delayed enhancement as assessed by MRI, is highly variable among patients and has strong prognostic relevance beyond left and right ventricular function for the occurrence of cardiovascular events. Thus, a more personalised risk stratification is clearly attainable. Less is known in subjects with prediabetes or pre-hypertensive stages of the disease, although there is early evidence that prediabetes is associated with increased subclinical disease burden, such as carotid plaque volume and arterial stiffness by ultrasound, changes of left ventricular function by echocardiography, or findings on native or contrast-enhanced cardiac MRI (Fig. 8). However, these observations lack a prospective design or reference group, comprise significant selection bias or are not generalisable to a preventive setting [17]. In order to confirm these early findings and establish their role in improving clinical care, further cross-sectional and longitudinal research in specific cohorts of patients is strongly warranted [17]. This may include prospective or nested cohort studies and randomised diagnostic trials [17]. As such, medical imaging may also add to the understanding of the relationship between changes in the phenotype and the individual predisposition for metabolic and cardiovascular diseases or the interplay with neurovascular diseases from a pathophysiology point of view. Accurate MRI evaluation of fat deposits within the liver and pancreas are also critical hallmarks of the metabolic syndrome.

## Challenge 5: Making Europe the world leader in machine learning and artificial intelligence in medical imaging by exploiting existing data and expertise to implement digital solutions after rigorous clinical validation

### Machine learning and artificial intelligence can substantially improve disease diagnostics and prediction

Medical image data has been accumulating exponentially since the introduction of digital radiology in the 1990s. For this reason, being already completely computerised, radiology is bound to be the first of all medical specialties embracing the use of machine-learning and artificial intelligence. By 2020, global medical data is expected to double every 73 days, and on average, each person generates one petabyte of health-related data across his or her lifetime [18]. Medical images are a substantial portion of all medical data, estimated to amount to 30% of all data storage [19]. The amount of data being collected exceeds our capacity to interpret them, especially when integrating multiple modalities from vastly different fields such as radiology, metabolomics and genetics.

Available medical imaging data can be a highly valuable resource for research on diagnostics, epidemiology and drug development. This has led to a higher interest in the development of data-driven models based on machine learning (ML) and artificial intelligence (AI) [18]. Deep learning, based on artificial neural networks, emerged in recent years as a powerful tool for ML and promises to reshape the future of AI. Increasing computational power allows ML, and AI in general, to more accurately identify and generate semantic interpretations from medical imaging data, supporting diagnoses.

Interpreting images can be highly subjective. ML and AI can be used to interpret images more consistently, as well as document metadata and perform data entry. ML techniques not only have significant potential to improve diagnoses from medical images, but may also improve disease prediction, decision-making, treatment planning and treatment response predictions [20].

Currently, ML and AI in medical imaging are not being used to their full extent. Beyond supporting diagnoses, ML and AI can reduce cost by cutting scan times, automating post-processing and reducing computation times for model-based image interpretation. Furthermore, ML and AI can accelerate drug discovery and leverage data from multiple sources (including sources other than medical images) for better care coordination.

### Examples addressing Challenge 5

#### Example 9: Identifying novel phenotypes in lung diseases

**Fig. 9:**
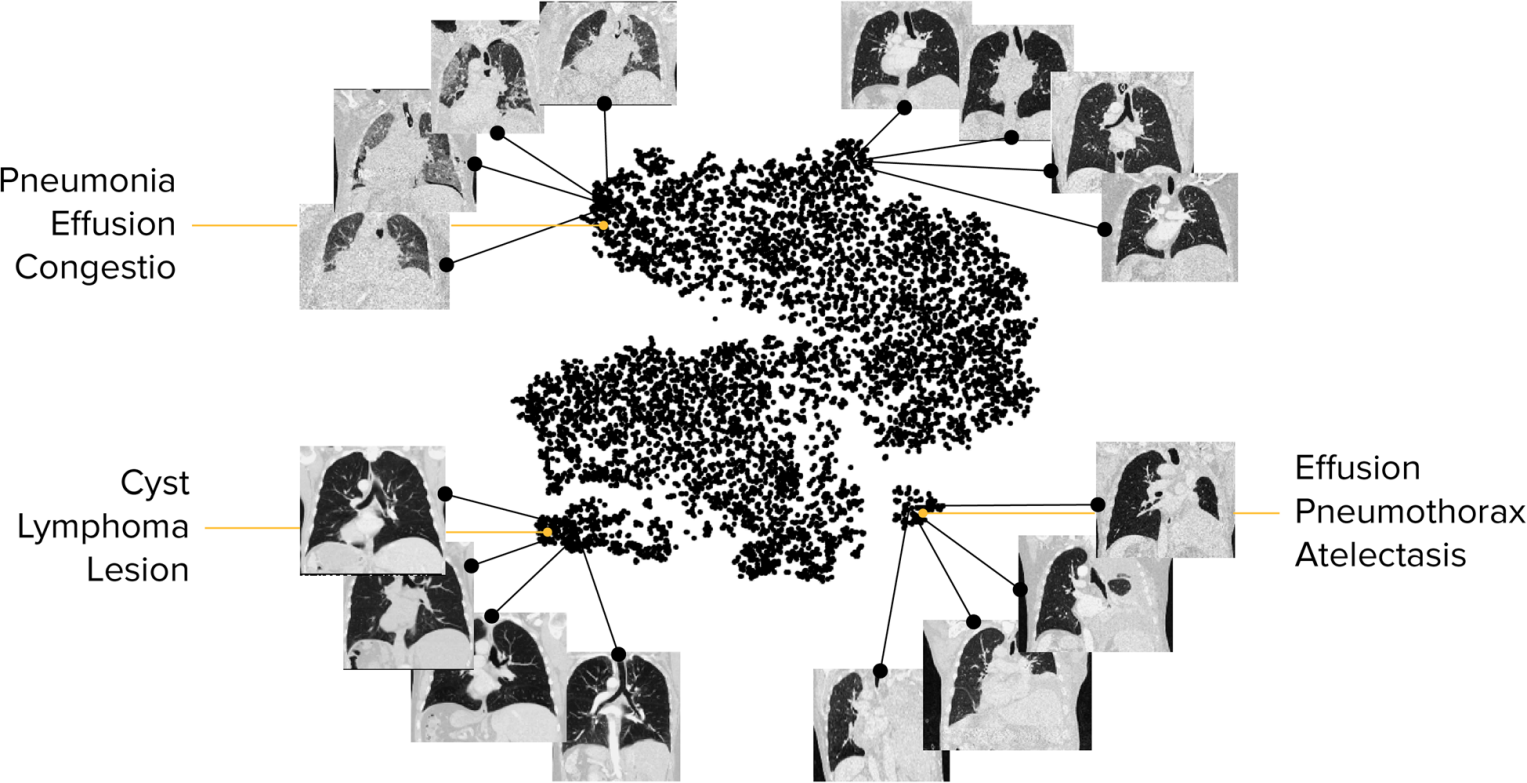
The landscape of lung disease patients based on their CT image data. The distribution illustrates clusters of patients with similar imaging characteristics confirmed in reported findings. It is a step towards the identification of phenotypes in large-scale medical imaging data. [24]

A key ability of ML is the exploitation of complex features drawn from multiple modalities and across the entire patient. Current finding and diagnostic categories in lung diseases such as fibrosis are lacking in terms of repeatability and their ability to predict outcome and treatment response. Machine learning can contribute by identifying those patterns that can be extracted with high stability and reliability from clinical imaging data (Fig. 9). It can connect these measurements with patient information such as smoking history, or age. Finally, it can mine the resulting descriptions for reliable predictors linking current patient status with outcome and risk that is relevant for individual treatment decisions to identify predictive markers. Initial results point to the power of machine learning in this direction, and we expect dramatic changes in both the characteristics we use for prognosis as well as the way we use them to treat an individual through the emerging integration of machine learning in the development of diagnostic tools, and the day-to-day clinical decision support.

#### Example 10: Automation of detection and measurements

**Fig. 10:**
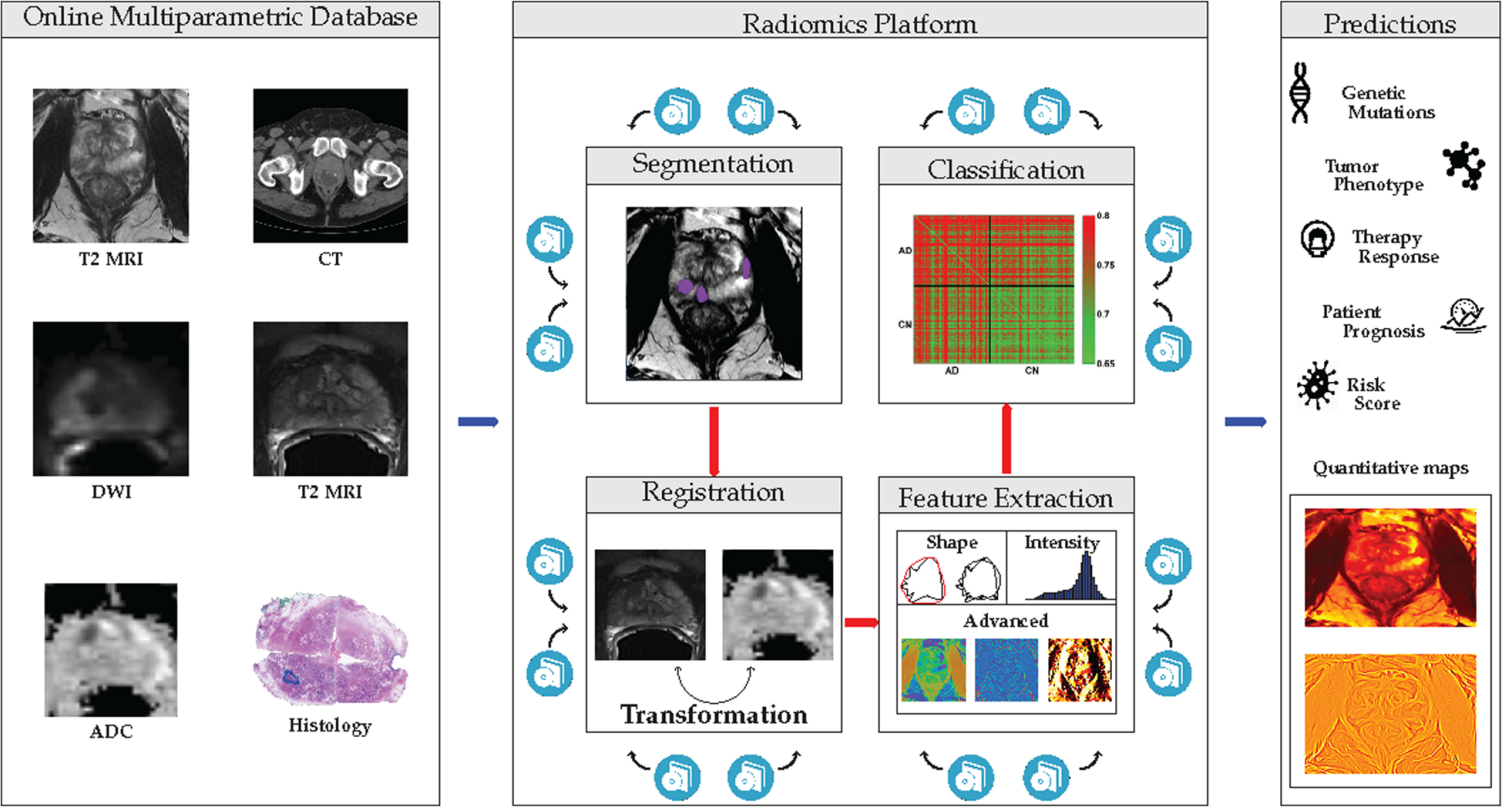
Radiomics pipeline, with the aim to link imaging features to clinically relevant parameters such as tumour subtype, patient prognosis and therapy outcome prediction (Courtesy Martijn Starmans and Stefan Klein, Rotterdam)

A wealth of known markers are tedious and time-consuming to extract from clinical imaging data. The widening gap between the rapidly increasing amounts of imaging data acquired during clinical routine, and the available number of radiologists who can read images, requires an increase of efficiency, and the delegation of tasks to machine learning-based algorithms. While the expert focuses on integrating individual measurements into a comprehensive assessment of the patient, the machine can reduce the time needed to screen hundreds of image slices. Examples comprising vast amounts of imaging data in need of analysis to improve disease diagnosis and prediction include the detection of nodules in lung imaging, polyps in CT-colonography, measurement of brain structures in neurological disease and evaluation of tumour characteristics for diagnosis and therapy outcome prediction (Fig. 10).

## Overcoming challenges in healthcare through biomedical imaging research

The EIBIR Strategic Research Agenda for Biomedical Imaging identifies research areas critical to overcoming current challenges in healthcare:

- Innovative biomedical imaging plays a key role in field of personalised medicine as it addresses the current needs for individualised prevention, treatment, therapy response monitoring and image-guided surgery. In the area of disease prevention and therapy monitoring, the use of non-invasive biomarkers will lead to improved patient outcome, therapy prediction and monitoring.
- Innovative diagnostic imaging technologies and procedures provide information about disease characteristics and, coupled with biological, genetic and -omics data, will contribute to an individualised diagnosis and consecutive targeted therapy approach. In the emerging field of theranostics, imaging tools together with therapeutic agents allow for tailored therapeutic interventions and the selection of most effective treatments.
- In the area of prenatal monitoring, the use of advanced imaging technologies with higher diagnostic accuracy will ensure the early detection of malfunctions or disease and benefit maternal and foetal health providing a healthy start in life.
- The use of biomedical imaging for diagnosis and management of lifestyle-induced diseases followed by lifestyle changes will help to prevent disease development.
- The advanced application of Artificial Intelligence and Machine Learning in imaging data will help to improve image interpretation and lead to better disease prediction, decision making and treatment planning.

In summary, biomedical imaging has the proven potential to address the major challenges in healthcare and to contribute to the wellbeing of European citizens and patients.

To realise this potential, investments in biomedical imaging research are needed to enable the translation of innovative solutions into clinical practice. Moreover, the appropriate funding will ensure that the European research community stays in the forefront of medical science.

Decision makers are invited to consider this document in the definition of research topics strategically recommendable for financing in the next decades.

## Supporting societies

This Strategic Research Agenda for Biomedical Imaging was developed by EIBIR with the support and input of its shareholder societies:

### European Society of Radiology (ESR)

The ESR is a non-profit organisation representing the general interests of radiology in Europe (www.myesr.org). The aims of ESR are to serve the healthcare needs of the general public through the support of science, teaching and research and the quality of service in the field of radiology as well as the promotion and coordination of the scientific, philanthropic, intellectual and professional activities of radiology in all European countries. The ESR has over 80,800 individual members as well as 109 member societies of which 47 are European national radiology societies, 15 are European radiological subspecialty societies and European allied sciences and 47 non-European national radiology societies.

### Cardiovascular and Interventional Radiological Society of Europe (CIRSE)

CIRSE is a non-profit, educational and scientific association aiming to improve patient care through the support of teaching, science, research and clinical practice in the field of cardiovascular and interventional radiology (IR) (www.cirse.org). CIRSE aims to support education and further research in IR, as well as ensuring excellent patient safety and timely access to IR therapies. CIRSE also actively collaborates with and supports other scientific, educational, governmental, professional, national and international organisations considered useful to the aims of the Society.

### European Coordination Committee of the Radiological, Electromedical and Healthcare IT Industry (COCIR)

COCIR is the European Trade Association representing the medical imaging, radiotherapy, health ICT and electromedical industries (www.cocir.org). COCIR is a non-profit association and provides a wide range of services on regulatory, technical, market intelligence, environmental, standardisation, international and legal affairs. COCIR promotes harmonisation of regulatory frameworks, supported by state-of-the-art international standards. Its industry provides safe and high-quality products and services, which contribute to reducing health inequalities and enhance cost efficiency in healthcare systems.

### European Association of Nuclear Medicine (EANM)

EANM is the umbrella organisation representing nuclear medicine in Europe and represents 40 National Member Societies, approximately 3200 individual members and around 30,000 professionals working in nuclear medicine in Europe (www.eanm.org). EANM aims to advance science and education in nuclear medicine for the benefit of public health, relating to the diagnosis, treatment, research and prevention of diseases through the use of unsealed radioactive substances and the properties of stable nuclides in medicine, throughout Europe.

### European Federation of Organisations for Medical Physics (EFOMP)

The EFOMP serves as an umbrella organisation representing 34 national member and affiliated organisations of more than 8100 physicists and engineers working in the field of medical physics in Europe (www.efomp.org). EFOMP aims to harmonise and advance medical physics in both its professional clinical and scientific expression throughout Europe by bringing about and maintaining systematic exchange of professional and scientific information, through the formulation of common policies, and by promoting education and training programmes.

### European Federation of Radiographer Societies (EFRS)

The EFRS is a non-profit umbrella organisation representing 39 professional societies and 60 educational institutions representing over 100,000 radiographers across Europe (www.efrs.eu). The aims of the EFRS are to represent, promote and develop the profession of radiography in Europe, across medical imaging, nuclear medicine and radiotherapy areas of radiography practice.

### European Organisation for Research and Treatment of Cancer (EORTC)

EORTC is an independent, non-profit cancer research organisation, with the mission to coordinate and conduct international translational and clinical research to improve the standard of cancer treatment for patients (www.eortc.org). EORTC aims ultimately to increase people’s survival and quality of life by testing new therapeutic strategies based on existing drugs, surgery and radiotherapy and also helps develop new drugs and approaches in partnership with the pharmaceutical industry and in patients’ best interests.

### European Society for Magnetic Resonance in Medicine and Biology (ESMRMB)

ESMRMB is a non-profit society, which aims to support educational activities and research in the widest sense in the field defined by the society’s name (www.esmrmb.org). The ESMRMB is open to physicians, engineers, scientists and other individuals who are interested in the developments or the introduction of magnetic resonance techniques in the fields of medicine and biology.

### European Society of Paediatric Radiology (ESPR)

ESPR aims to organise and bring together physicians involved in the field of paediatric imaging, to contribute to the progress of paediatric imaging particularly within but also outside Europe, to encourage training and education with other branches of medical imaging and paediatrics in clinical, scientific, education and research fields (www.espr.org).

### European Society for Radiotherapy and Oncology (ESTRO)

ESTRO is a non-profit scientific organisation representing radiation oncologists, medical physicists, radiobiologists and radiation therapists with over 5000 members both within and outside Europe (www.estro.org). ESTRO aims to foster the role of radiation oncology in order to improve patient care in the multimodality treatment of cancer by promoting innovation, research and dissemination of science through its congresses, special meetings, educational courses and publications.

### European Society of Medical Imaging Informatics (EuSOMII)

EuSoMII is a professional healthcare organisation that provides its members and the radiological community with up-to-date information on the latest innovations and achievements in medical imaging informatics by supporting education, research and events related to the top-tier software in radiology (www.eusomii.pro).
